# Chrysanthemum *WRKY15-1* promotes resistance to *Puccinia horiana* Henn. via the salicylic acid signaling pathway

**DOI:** 10.1038/s41438-020-00436-4

**Published:** 2021-01-01

**Authors:** Mengmeng Bi, Xueying Li, Xin Yan, Di Liu, Ge Gao, Pengfang Zhu, Hongyu Mao

**Affiliations:** 1 College of Forestry, Shenyang Agricultural University, Shenyang, 110866 China; 2 Key Laboratory of Forest Tree Genetics, Breeding and Cultivation of Liaoning Province, Shenyang, 110866 China

**Keywords:** Biotic, Genetic engineering

## Abstract

Chrysanthemum white rust disease, which is caused by the fungus *Puccinia horiana* Henn., severely reduces the ornamental quality and yield chrysanthemum. WRKY transcription factors function in the disease-resistance response in a variety of plants; however, it is unclear whether members of this family improve resistance to white rust disease in chrysanthemum. In this study, using PCR, we isolated a *WRKY15* homologous gene, *CmWRKY15-1*, from the resistant chrysanthemum cultivar C029. Real-time quantitative PCR (RT-qPCR) revealed that *CmWRKY15-1* exhibited differential expression patterns between the immune cultivar C029 and the susceptible cultivar Jinba upon *P. horiana* infection. In addition, salicylic acid (SA) treatment strongly induced *CmWRKY15-1* expression. Overexpression of *CmWRKY15-1* in the chrysanthemum-susceptible cultivar Jinba increased tolerance to *P. horiana* infection. Conversely, silencing *CmWRKY15-1* via RNA interference (RNAi) in C029 increased sensitivity to *P. horiana* infection. We also determined that *P. horiana* infection increased both the endogenous SA content and the expression of salicylic acid biosynthesis genes in *CmWRKY15-1*-overexpressing plants, whereas *CmWRKY15-1* RNAi plants exhibited the opposite effects under the same conditions. Finally, the transcript levels of pathogenesis-related (*PR*) genes involved in the SA pathway were positively associated with *CmWRKY15-1* expression levels. Our results demonstrated that *CmWRKY15-1* plays an important role in the resistance of chrysanthemum to *P. horiana* by influencing SA signaling.

## Introduction

When plants are exposed to external pathogens through stomata or wounds, the first line of defense is triggered: pathogen-associated molecular pattern-triggered immunity (PTI). PTI prevents several types of pathogens from entering cells by triggering reactive oxygen species bursts and callose deposition. Once they are activated, pattern recognition receptors on the cell membrane in turn induce the activity of related kinases and the activation of downstream signal transduction pathways^1–3^. Signals in these pathways culminate in the nucleus, where they mediate a series of resistance-related reactions, such as the transcription and translation of disease-related proteins, the expression of various transcription factors, and microRNA synthesis^4^. The second plant line of defense is effector-triggered immunity (ETI), which inhibits the growth and spread of pathogenic bacteria via the process of programmed cell death. WRKY transcription factors play critical roles in both PTI and ETI.

Members of the WRKY transcription factor family, one of the largest transcription factor protein families in plants, are involved in biotic stress tolerance and participate in both signal transduction pathways and gene expression regulation. WRKY proteins contain two highly conserved domains: the WRKY domain, with the amino acid motif WRKYGQK, at their N-terminus and a novel C_2_–H_2_ or C_2_HC type zinc-finger motif at their C-terminus^5–7^. Based on their motif arrangements, WRKY proteins can be classified into four groups^8^. Moreover, WRKY transcription factors can specifically bind to the W-box element (TTGACC/T) in the promoters of various downstream biotic stress-related genes to regulate their transcription and enhance plant defense^9^. Arabidopsis *WRKY* genes such as *WRKY18*, *WRKY28*, *WRKY52*, and *WRKY33* have been shown to enhance resistance to the several pathogenic bacterial species, including *Pseudomonas syringae*, *Sclerotinia sclerotiorum*, *Ralstonia solanacearum*, and *Botrytis cinerea*^10–13^. Similarly, the wheat (*Triticum aestivum*) genes *TaWRKY45* and *TaWRKY70* promote resistance to stripe rust and leaf rust in the cultivar Xiaoyan 6^14,15^. WRKY transcription factors regulate a variety of signaling networks involved in plant disease-resistance-related responses, such as networks involving abscisic acid (ABA), salicylic acid (SA), jasmonic acid/ethylene (JA/ET), mitogen-activated protein kinases (MAPKs), and histone deacetylases^16,17^. The expression of *WRKY* genes during plant defense responses closely parallels that of genes involved in these signaling pathways^18^. In Arabidopsis (*Arabidopsis thaliana*), *WRKY18*, *WRKY38*, *WRKY54*, and *WRKY66* participate in SA signal transduction^19^. *AtWRKY18* enhances the resistance of transgenic *A*. *thaliana* to *P*. *syringae* by activating *PR* gene expression in the SA pathway^20^. WRKY transcription factors function in conjunction with *PR1-1* and *PR2* in the SA signaling pathway to regulate plant resistance to anthracnose; moreover, exogenous SA application reduces the disease index of banana (*Musa acuminata*) infected with anthracnose^21^. Overexpression of *CsWRKY50* in cucumber (*Cucumis sativus*) enhances plant resistance to the fungal pathogen *Psilocybe cubensis* and upregulates the transcript levels of several phytohormone-related defense genes, including SA- and JA-responsive genes and SA biosynthesis genes^22^.

*Chrysanthemum morifolium* is a popular ornamental plant species worldwide with great economic and cultural value. Chrysanthemum white rust caused by the fungus *Puccinia* *horiana* severely affects chrysanthemum cultivation. Once the disease takes hold, it tends to spread extensively, which causes economic losses and hinders production. In addition to reducing chrysanthemum ornamental quality and yield, *P. horiana* may even lead to plant death. Although the roles of WRKY transcription factors in the resistance mechanism of plants have been extensively studied, it is unclear whether members of the WRKY family in chrysanthemum contribute to the response to chrysanthemum white rust infection. Here, we identified a gene whose expression is significantly induced, *CmWRKY15-1*, from transcriptomic data collected upon *P. horiana* infection^23^. We explored the role of *CmWRKY15-1* in the regulation of chrysanthemum resistance to *P. horiana* infection and established that this gene improves resistance to white rust disease either directly or indirectly via the SA-mediated disease-resistance signaling pathway.

## Results

### Isolation and Sequence Analysis of *CmWRKY15-1*

**Fig. 1:**
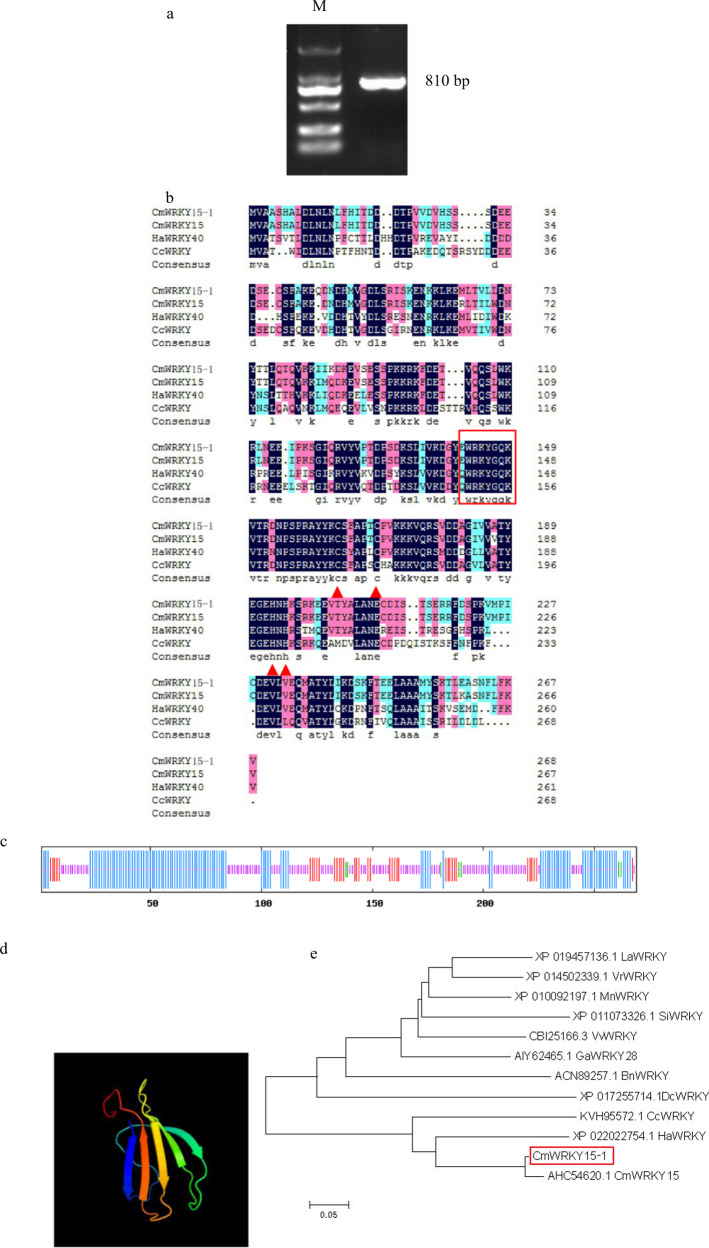
Bioinformatic analysis of *CmWRKY15-1*. **a** PCR amplification product of *CmWRKY15-1*. **b** Multiple amino acid sequence alignment between CmWRKY15-1 and several related WRKY proteins from the Asteraceae. The box indicates the WRKYGQK heptapeptide sequence; the triangle indicates the zinc-finger motif. **c** Secondary structure of the predicted CmWRKY15-1. Blue, alpha-helix; red, folding and extending chain; green, beta-turn; purple, random coil. **d** Three-dimensional structure of predicted CmWRKY15-1. **e** Phylogenetic analysis of CmWRKY15-1. M Marker, La *Lupinus angustifolius*; Vr *Vigna radiata* var. *radiata*; Mn *Morus notabilis*; Si *Sesamum indicum*; Vv *Vitis vinifera*; Ga *Gossypium aridum*; Bn *Brassica napus*; Dc *Daucus carota* subsp. *Sativus*; Cc *Cynara cardunculus* L.; Ha *Helianthus annuus* L.; Cm *Chrysanthemum morifolium*.

We isolated an 810-bp full-length cDNA encoding a predicted protein of 269 amino acids from C029 (Fig. 1a) and analyzed the sequence via BLAST searches against published sequences in GenBank. The nucleotide sequence was 98% similar to that of the previously published chrysanthemum gene *WRKY15*; therefore, we named this gene *CmWRKY15-1*. The predicted CmWRKY15-1 protein contains a typical WRKY domain that contains a WRKYGQK motif distributed between amino acids 137 and 195 and a C_2_–H_2_ zinc-finger motif, both of which are hallmarks of class II WRKY transcription factors (Fig. 1b). The protein contains 46 negatively charged residues and 40 positively charged residues. The instability coefficient of CmWRKY15-1 is 49.68, while its average hydrophobicity is −0.647. These results indicated that CmWRKY15-1 is likely to be an unstable and hydrophilic protein.

Based on predictions of protein structure and phylogenetic analysis of CmWRKY15-1, we determined the secondary structure of CmWRKY15-1 using SOPMA (http://npsa-pbil.ibcp.fr/). Of the 269 amino acids present in CmWRKY15-1, 118 were part of alpha-helices, 37 were part of folded-form extension chains, 7 were part of beta-turns, and 107 were part of random coils, accounting for 43.9, 13.75, 2.6, and 39.8% of the protein, respectively (Fig. 1c). Figure 1d depicts the three-dimensional structure of the predicted CmWRKY15-1 protein. We next compared the structure of CmWRKY15-1 to similar protein structures reported in the Phyre database to predict its tertiary structure. The CmWRKY15-1 protein was 97% similar to CmWRKY15 and 68 and 62% similar, respectively, to other WRKY proteins from two other Asteraceae species, sunflower (*Helianthus annuus*) and artichoke thistle (*Cynara cardunculus*). CmWRKY15-1 appears to be most closely related to WRKY proteins from rapeseed (*Brassica napus*) and carrot (*Daucus carota* subsp. *sativus*) (Fig. 1e).

### Expression Profiles of *CmWRKY15-1* Under Stress Treatments

**Fig. 2:**
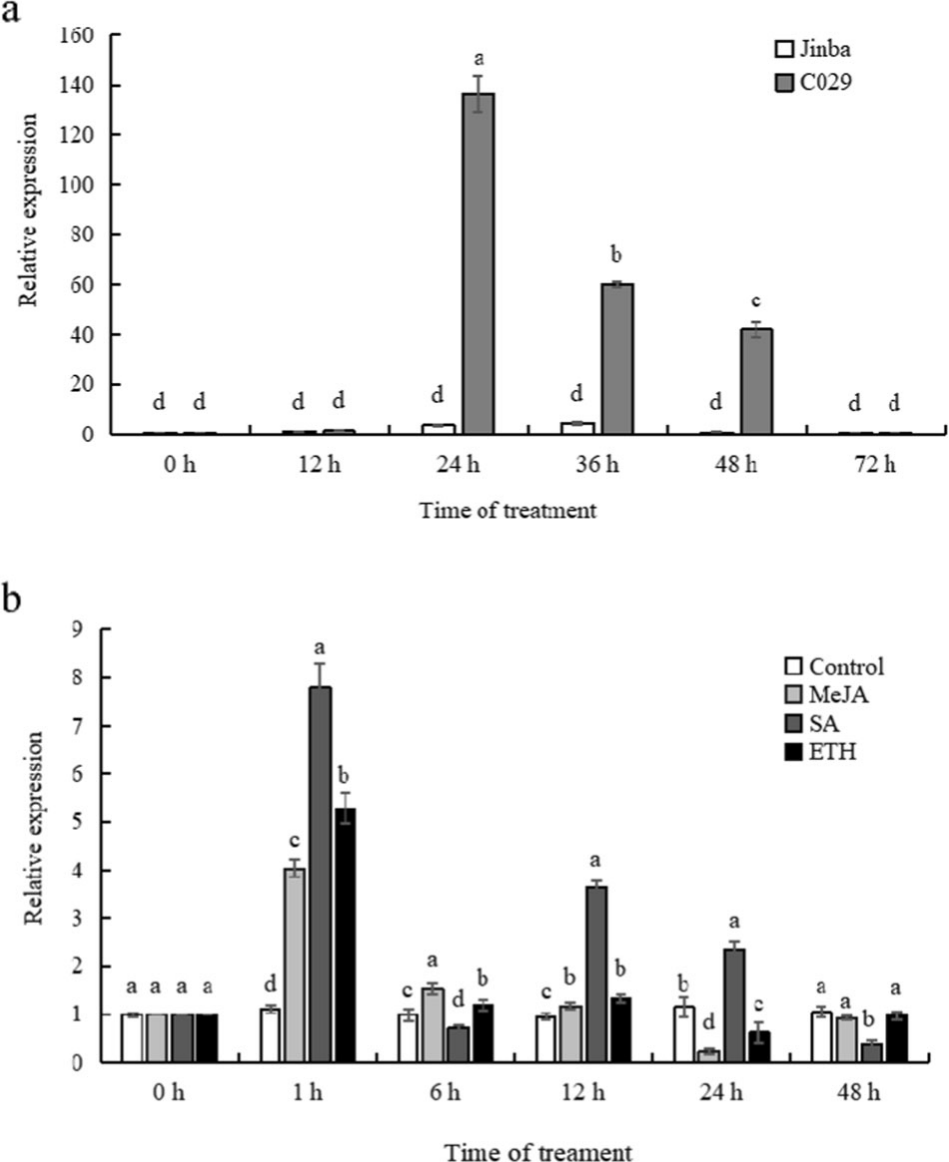
Expression patterns of *CmWRKY15-1* under various treatments. **a** *CmWRKY15-1* transcript levels in chrysanthemum leaves during infection with *P. horiana* in C029 and Jinba. **b** *CmWRKY15-1* expression levels after water and exogenous hormone treatments. SA, 0.1 mM salicylic acid; MeJA, 50 μM methyl jasmonate; ETH, 0.5 g L^−1^ ethephon (ETH) in C029. The error bars indicate the standard deviations of three replicates. The different letters mean significant differences according Duncan’s multiple range test at *p* < 0.05; the same scheme applies below.

To explore the potential role of *CmWRKY15-1* in chrysanthemum, we first determined the expression patterns of *CmWRKY15-1* during a 72 h inoculation treatment in the resistant cultivar C029 and the susceptible cultivar Jinba by RT-qPCR. *CmWRKY15-1* transcript levels were high from 24 to 48 h after inoculation (Fig. 2a). In addition, *CmWRKY15-1* expression was generally much higher in the resistant cultivar C029 than in the susceptible cultivar Jinba. We also analyzed *CmWRKY15-1* expression following exogenous application of phytohormones in C029 and observed an ~7.8-fold increase in *CmWRKY15-1* transcript levels after 1 h of SA treatment, while JA or ET resulted in more modest increases (4.0-fold for JA and 5.3-fold for ETH), although all the tested phytohormones did increase *CmWRKY15-1* transcript levels (Fig. 2b). These results suggest that, during infection with *P. horiana*, a temporal increase in *CmWRKY15-1* expression in the leaves may be associated with modulating resistance to *P. horiana* and that *CmWRKY15-1* expression is strongly induced by the phytohormone SA.

### Plasmid Construction and Generation of Transgenic Chrysanthemum

**Fig. 3:**
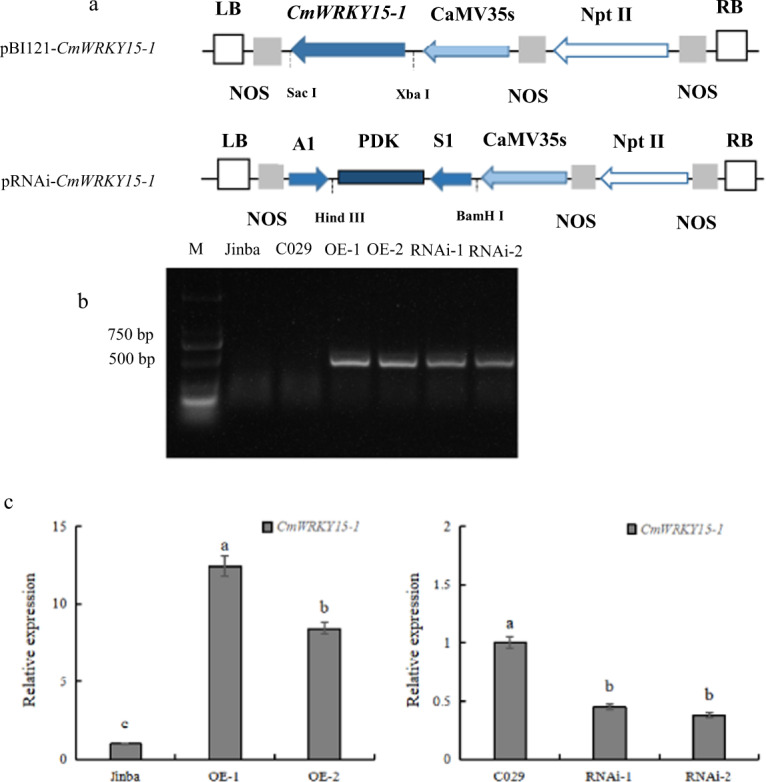
Acquisition of transgenic plants. **a** Diagram of the pBI121-*CmWRKY15-1* and pRNAi-*CmWRKY15-1* vectors. CaMV 35 S, promoter; NOS, nopaline synthase terminator; *nptII*, neomycin phosphotransferase gene; *CmWRKY15-1*, target fragment; LB and RB, left and right borders of the T-DNA; SacI, XbaI, BamHI, and HindIII, cloning sites; A1, antisense fragment; S1, sense fragment; PDK, intron. **b** PCR-based analysis of kanamycin-resistant transgenic plants. M, DNA ladder (DL 2000); OE-1 and OE-2, overexpression transgenic plants; RNA1 and RNA2, RNA interference transgenic plants. **c** Relative expression levels of *CmWRKY15-1*.

To investigate whether *CmWRKY15-1* plays a role in controlling resistance to *P. horiana* infection, we generated an overexpression vector based on the pBI121 vector, which contains the selectable marker gene *nptII*, affording kanamycin resistance (Fig. 3a). We generated overexpression (OE) and silenced (RNA interference [RNAi]) *CmWRKY15-1* chrysanthemum plants, obtaining 12 *T*_0_ clones of *CmWRKY15-1*-OE plants and 18 *T*_0_ clones of *CmWRKY15-1*-RNAi plants. We selected two representative and independent positive plants for each of the transgenic lines (OE-1, OE-2, RNAi-1, and RNAi-2) (Fig. 3b) to measure *CmWRKY15-1* transcript levels via RT-qPCR. Compared with untransformed wild-type (WT) plants, the OE lines exhibited 12- and 8-fold increases in *CmWRKY15-1* relative expression levels, and the RNAi plants presented reduced *CmWRKY15-1* transcript levels, with downregulation ranging from 55 to 62% (Fig. 3c). We then assessed the phenotypes of these plants in terms of their growth and development but observed no obvious differences in plant height, crown diameter, or leaf number for any of the transgenic plants relative to the wild type.

### Degree of Resistance to *P. horiana* Infection of Chrysanthemum Transgenic Plants

We next performed a pathogen infection test on all plant genotypes (the Jinba and C029 wild types, as well as OE-1, OE-2, RNAi-1, and RNAi-2) for 14 days. We treated 30–40 leaves per line and assessed their infection phenotypes, calculated their associated disease severity index **(**DSI), and then determined whether the plants were resistant or susceptible to *P. horiana*.

**Fig. 4:**
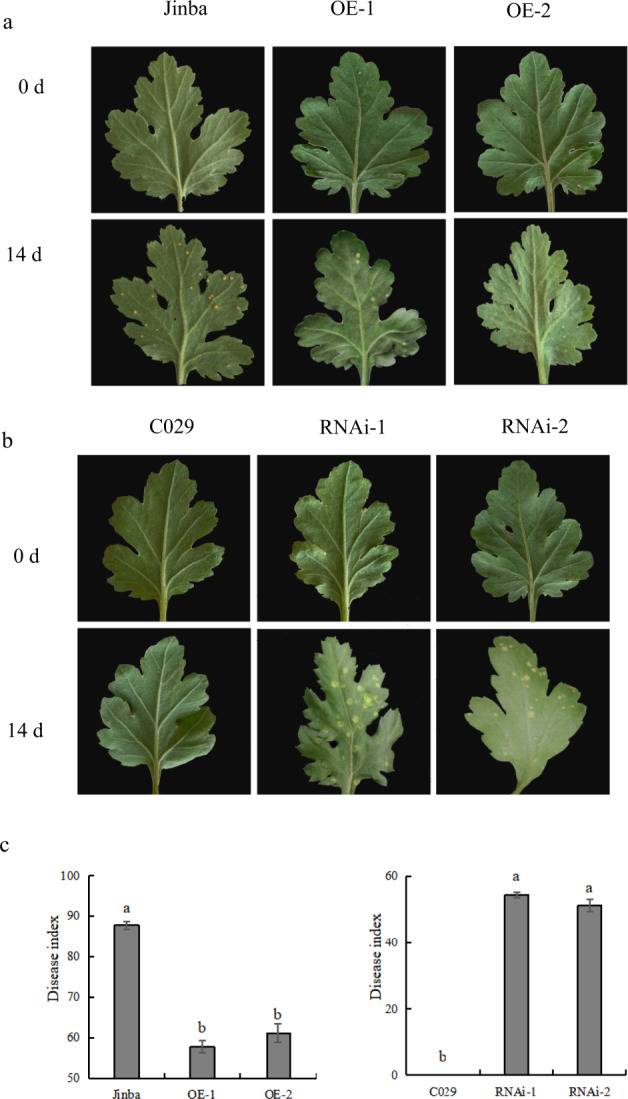
Phenotypes of plants in which *CmWRKY15-1* was overexpressed or silenced upon infection with *P. horiana*. **a**, **b** Leaf phenotypes at 2 weeks after infection. **c** Disease index of plants.

Immediately after inoculation, the leaves of all three line types (WT, OE, and RNAi) were similar. We observed discontinuous teliospores in the leaves of Jinba after 14 days, as well as clear white spots and some visible spores, which is consistent with expected symptoms for a susceptible plant (S). By contrast, the OE plants showed few white spots on their leaves, and some leaves had no sporozoites, even after 14 days (Fig. 4a), making them moderately resistant (MR). In addition, we evaluated the disease resistance of the RNAi lines. As shown in Fig. 4b, compared with the control plants, the *CmWRKY15-1*-silenced plants exhibited a higher sensitivity to *P. horiana* infection after 14 days, as evidenced by the low frequency of light macula on the leaf surface and the discontinuous teliospore heaps on the abaxial side of the leaf. The degree of infection in the RNAi plants was not as severe as that in Jinba, indicating an MR-resistance type. As expected, C029 showed immunity (I) to the fungus.

The DSI of the OE lines was considerably lower than that of Jinba (Fig. 4c). By contrast, the RNAi lines had a considerably higher DSI than their corresponding C029 wild-type plants did.

At 14 days after infection, we scored the number of plants with visible symptoms and quantified the disease index for Jinba (87.8%), OE-1 (57.8%), and OE-2 (61.1%). The disease index of the RNAi-1 and RNAi-2 plants was significantly higher than that of C029, reaching 54.4 and 51.1% of the index of the blank control group, respectively. These results indicated that *CmWRKY15-1* is involved in the disease resistance of chrysanthemum.

### Changes in SA Levels and Expression of SA Biosynthesis Genes

**Fig. 5:**
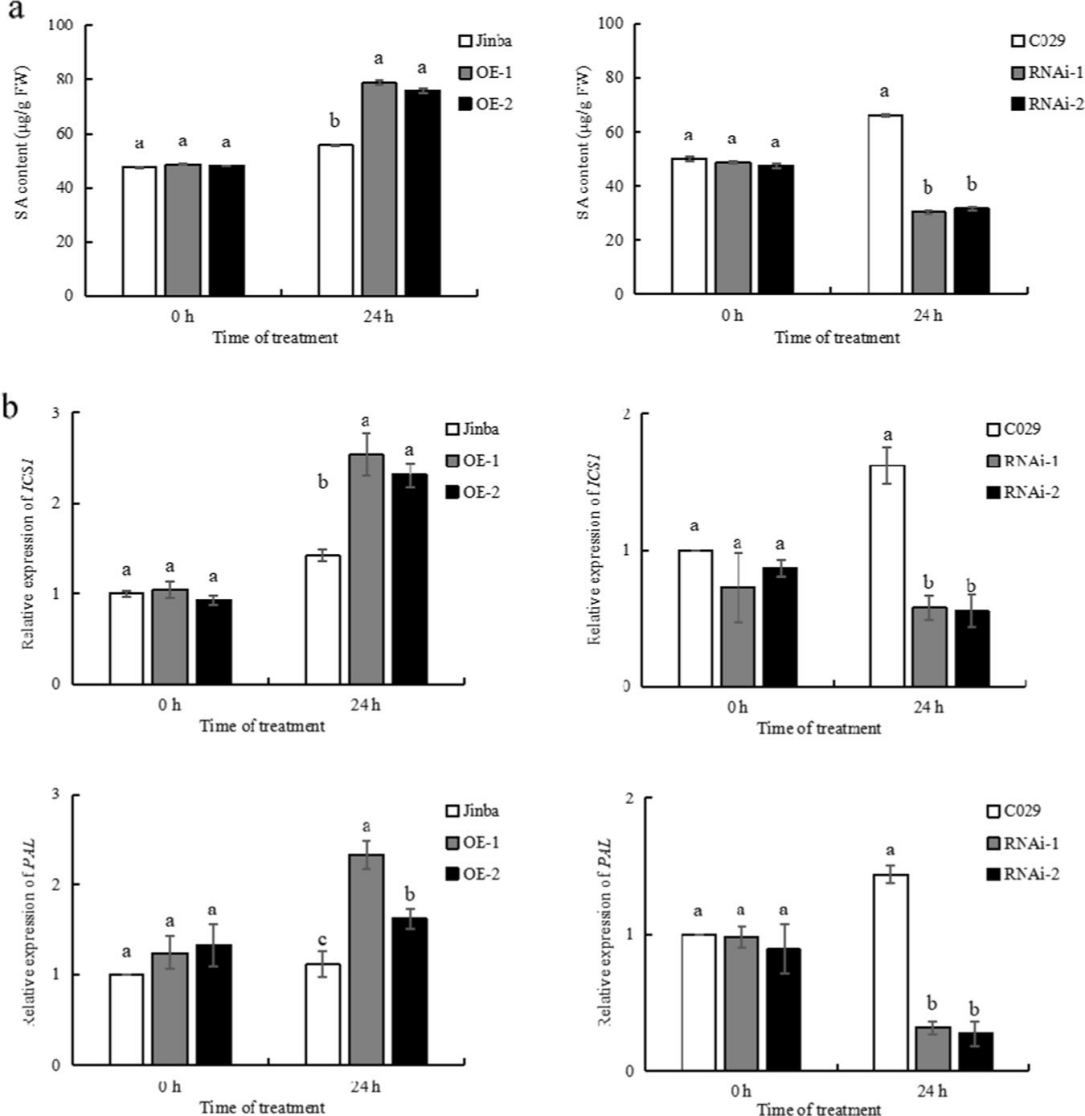
Endogenous hormone contents. **a** SA levels in transgenic plant leaves during 0–24 h after *P. horiana* infection. FW, fresh weight. **b** Expression analysis of the SA biosynthesis genes *ICS1* and *PAL*.

To investigate whether endogenous phytohormones might participate in plant resistance to *P. horiana* and whether *CmWRKY15-1* might be involved, we measured the levels of endogenous SA in the *CmWRKY15-1* transgenic plants and corresponding wild types at 24 h after infection with *P. horiana*. We discovered that *P. horiana* infection triggered an increase in SA levels in both the OE-1 and OE-2 lines, whereas SA levels were reduced in the RNAi lines relative to those in the WT (Fig. 5a). An analysis of cis-regulatory elements within the *CmWRKY15-1* promoter revealed a number of SA signal-response elements. We therefore speculated that *CmWRKY15-1* might be involved in SA signaling. To test this hypothesis, we analyzed the expression level of the key SA biosynthesis genes isochorismate synthase 1 (*ICS1*) and phenylalanine ammonia lyase (*PAL*) before and after *P. horiana* infection. We observed no significant differences in the transcript levels of *ICS1* or *PAL* between the noninfected transgenic plants and the WT (Fig. 5b). However, the *ICS1* transcript levels increased in the OE lines 24 h after inoculation. *PAL* expression followed the same trend, with higher levels in both OE lines relative to those in the control group. In contrast to those in the OE lines, the transcript levels of both the *ICS1* and *PAL* genes in the RNAi lines decreased 24 h after inoculation. Based on these results, we conclude that the changes in endogenous SA levels are closely related to *CmWRKY15-1* expression.

### *CmWRKY15-1* Regulates Pathogenesis-Related Genes Involved in the SA Signaling Pathway

**Fig. 6:**
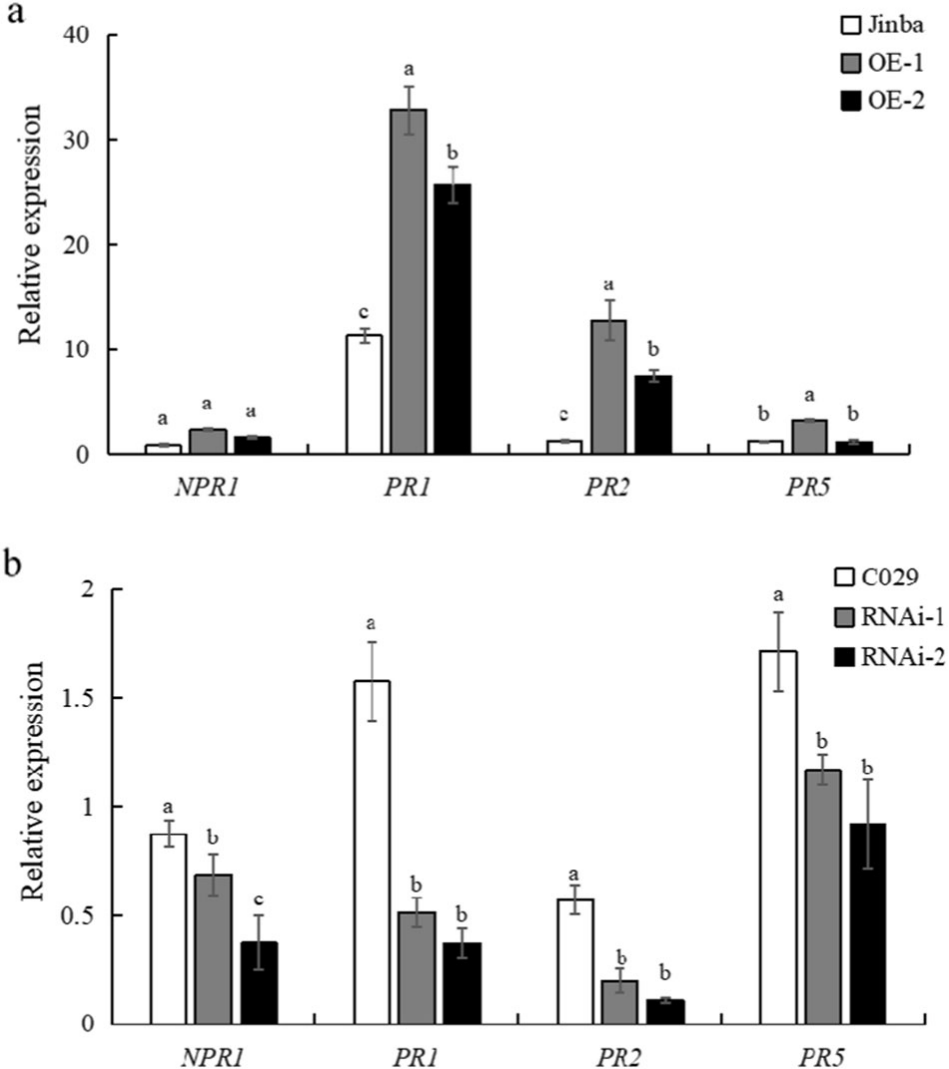
Expression of pathogenesis-related genes involved in the SA signaling pathway. **a** Pathogenesis-related gene expression in the control (Jinba) and overexpression plants. **b** Pathogenesis-related gene expression in the control (C029) and silenced plants.

To determine the role of SA and *CmWRKY15-1* in the regulation of chrysanthemum resistance to *P. horiana*, we tested whether *CmWRKY15-1* might directly regulate the expression of defense-related genes. Here, we used RT-qPCR to quantify the relative expression levels of the pathogenesis-related (*PR*) genes nonexpresser of PR genes 1 (*NPR1*), pathogenesis-related 1 (*PR1*), *PR2*, and *PR5*, which are SA marker genes. *PR1* expression was sharply upregulated in the OE lines after *P. horiana* infection compared to the expression in the infected wild-type controls (Fig. 6a). *PR2* responded similarly, as did *PR5*, at least in the OE-1 background. The expression of *NPR1* did not change significantly between the WT and OE lines. These results indicated that the resistance of transgenic chrysanthemum plants to *P. horiana* infection may be associated with the upregulated expression of defense-related genes. In agreement with this hypothesis, all defense-related genes showed reduced transcript levels in the RNAi lines following *P. horiana* infection relative to those in the wild type. Even *NPR1* transcript levels were significantly lower in the RNAi lines than in the WT, although the effect was not as pronounced as that of the other *PR* genes (Fig. 6b).

Collectively, these results indicate that *CmWRKY15-1* regulates the resistance of chrysanthemum to *P. horiana* infection by modulating the SA signaling pathway.

## Discussion

Multiple lines of evidence suggest that WRKY transcription factors play roles in regulating pathogen infection in plants^24–27^. To explore the function of WRKY transcription factors in chrysanthemum, we isolated a differentially expressed gene based on transcriptomic data of chrysanthemum treated with *P. horiana*. This gene, *CmWRKY15-1*, encodes a WRKY-type transcription factor, which we speculated was related to the regulation of disease resistance, and we tested the role of this gene in the regulation of disease responses.

Plant defense systems involve a complex signal regulatory network in which plant hormones such as SA, JA, and ethylene play a crucial role^28–30^. SA enhances plant resistance to pathogen attack, regulates defense responses to various pathogens, and increases the transcript levels of pathogenesis-related genes^31,32^. SA is synthesized mainly through two pathways: the *ICS* and *PAL* pathways^33^. The exact biosynthesis pathway varies among plant species. For example, in Arabidopsis and tobacco (*Nicotiana tabacum*), SA is synthesized by the *PAL* pathway. In soybean (*Glycine max*), both pathways contribute to SA biosynthesis^34^. The *ICS* pathway is generally considered to be the main source of continuous SA biosynthesis, while the *PAL* pathway synthesizes SA rapidly only in locally necrotic cells^35^. In our study, the expression of the *ICS* and *PAL* SA biosynthesis genes also significantly increased in the *CmWRKY15-1* OE lines after *P. horiana* infection (Fig. 5b), and their expression showed the opposite trend in the RNAi lines, which is consistent with the change in endogenous SA content measured across all transgenic plants. These results suggested that *CmWRKY15-1* overexpression may result in stronger defense responses through increased SA accumulation.

In chrysanthemum, previous studies have reported that a number of *WRKY* genes are induced or repressed by pathogens. *CmWRKY1*, *CmWRKY11*, and *CmWRKY15* are induced by *Alternaria tenuissima* inoculation^36^. *CmWRKY1*, *CmWRKY6*, and *CmWRKY8* are also induced by *Fusarium oxysporum*, whereas *CmWRKY4*, *CmWRKY8*, and *CmWRKY11* expression is significantly repressed by *P. horiana* infection. Among the 15 chrysanthemum *WRKY* genes (*CmWRKY1* to *CmWRKY15*), 11 are induced by SA; 3, ABA; and 4, MeJA. These high numbers suggest that these WRKYs may be involved in disease-resistance defense pathways. Here, *CmWRKY15-1* was induced by *P. horiana* infection in both susceptible and resistant chrysanthemum, and its expression level in resistant chrysanthemum was significantly higher than in susceptible chrysanthemum. In addition, *CmWRKY15-1* was also induced by the phytohormones SA, MeJA, and ET, with SA resulting in the strongest induction. Therefore, it is reasonable to hypothesize that *CmWRKY15-1* may participate in the disease resistance of chrysanthemum to *P. horiana* and that the disease-resistance process is mediated by phytohormone signals.

WRKY transcription factors often control, directly or indirectly, the expression of disease-resistance genes by activating signaling pathways^37^. To date, a variety of WRKY transcription factors have been shown to be involved in different phytohormone-dependent defense pathways. Sixteen *WRKY* genes are induced in response to *S*. *sclerotiorum* infection in rapeseed, some of which are involved in signaling pathways such as the SA and JA pathways^38^. The expression levels of *PR1* and plant defensin 1.2 (*PDF1*.*2*) increased 2.5- and 3.3-fold, respectively, in plants overexpressing rapeseed *WRKY33*, indicating that rapeseed *WRKY33* is involved in SA- and JA-mediated signaling pathways^39^. Mutation of Arabidopsis *WRKY33* results in decreased expression of the JA signaling-related gene *PDF1.2* and increased susceptibility to *B*. *cinerea*. Overexpression of Arabidopsis *WRKY33* decreases the expression level of *PR1* and increases plant susceptibility to *P*. *syringae*. Thus, the regulatory effects of Arabidopsis *WRKY33* on defense pathways against *B. cinerea* and *P*. *syringae* are antagonistic^40^.

SA signaling is largely involved in the response to vegetative pathogens of living organisms, such as *Oidium neolycopersici* and *Hyaloperonospora parasitica*^41^, whereas JA/ET signals are targeted toward necrotizing pathogens^42^. We measured expression changes in the JA-ET pathway-related gene *PDF1.2* when WT and transgenic plants were inoculated with *P. horiana* but observed no differences in either genotype. Simultaneously, we used RT-qPCR to analyze the expression of the PR genes *NPR1*, *PR1*, *PR2*, and *PR5* and detected significant changes in their expression in the transgenic plants but not in the wild type. The *CmWRKY15-1*-overexpressing plants showed enhanced resistance to *P. horiana* infection, likely due to the upregulation of *PR* genes. Conversely, silencing *CmWRKY15-1* resulted in significantly decreased *NPR1*, *PR1*, *PR2*, and *PR5* transcript levels. However, *NPR1* expression was not significantly altered in the overexpression lines. This discrepancy may be due to a signaling interaction between NPR1 and WRKY transcription factors. Several studies have shown that NPR1 activates the expression of downstream *WRKY* genes by interacting with a TGA-type transcription factor at their promoters, thereby promoting the expression of downstream disease-resistance genes^43–45^. The promoter region of *NPR1* does contain a W-box, which indicates that *NPR1* itself may be regulated by one or more WRKY transcription factors^46^. These results further suggest that *CmWRKY15-1* acts as a positive regulator of chrysanthemum resistance to *P. horiana* via the SA signaling pathway. Our future research will focus on how *CmWRKY15-1* transcription factors interact with *PR* genes. Our results provide a solid theoretical basis for breeding chrysanthemum varieties that are resistant to chrysanthemum white rust and serve as a reference point for discovering functional genes involved in the SA signaling pathway.

## Materials and methods

### Plant materials and growth conditions

All experiments were conducted at the Forestry College of Shenyang Agricultural University, Shenyang, China, from 2018 to 2020. The chrysanthemum resistant cultivar C029 and susceptible cultivar Jinba were provided by the flower base of the Forestry College of Shenyang Agricultural University.

Seedlings at the 6- to 8-leaf stage were grown in a potting soil mixture and placed in a greenhouse under fluorescent lights for 2 weeks at 25 ± 3 °C.

### Pathogen culture

We collected teliospores of *P. horiana* from the abaxial side of chrysanthemum leaves infected with white rust and placed the teliospores in 1 mL of sterile water. We adjusted the concentration of the teliospores to 40–60 per field of vision under BA400 microscope (Motic, Xiamen). We removed a drop of teliospore suspension with a sterile straw and then dropped the suspension onto a glass substrate that was placed on a U-shaped rod in a culture dish covered with wet filter paper. The teliospores were allowed to germinate under a temperature of 18–21 °C for 24 h, after which we resuspended the germinated spore suspension in sterile water with 0.05% (w/v) Tween 20 pH (4–6.5). We then sprayed the abaxial side of the plant leaves evenly with the solution before covering the plants with plastic film and moving them into the dark and high humidity. After 16–24 h, we transferred the infected plants to new growth conditions of 17 °C and 50% humidity. The fungi started to release basidiospores, which germinated within 3 h Basidiospores invade leaf surfaces^47–51^.

### RNA isolation and cDNA synthesis

We extracted the total RNA from the leaves of C029 using an RNA prep Pure Plant Kit (Tiangen, Beijing). We subsequently synthesized first-strand cDNAs using the Prime Script II 1^st^ Strand cDNA Synthesis Kit following the manufacturer’s protocol (Takara, Japan).

### Isolation and sequencing analysis of *CmWRKY15-1*

We amplified the coding sequence of Cm*WRKY15-1* using *CmWRKY15-1* forward (F) and reverse (R) primers. The PCR program was as follows: denaturation at 94 °C for 5 min; 35 cycles of 30 s at 94 °C, 30 s at 57 °C, and 2 min at 72 °C; and a final extension at 72 °C for 10 min. We purified the PCR product and subcloned it using a p-TOPO Zero Background Kit (Aidlab, Beijing). We transformed the ligation reaction into *Escherichia coli* DH5α (Aidlab) and identified the clones harboring the inserts for subsequent sequencing.

### Bioinformatic analysis

We analyzed the DNA and deduced the protein sequences from *CmWRKY15-1* with DNAMAN software. We carried out a phylogenetic analysis of CmWRKY15-1 and related WRKY proteins via MEGA 5.0. We used the online tool ExPASy (http://expasy.org) to predict the physicochemical properties of CmWRKY15-1 and the SOPMA tool (http://npsa-pbil.ibcp.fr/) and Phyre^2^ database to analyze the predicted protein structure.

### Analysis of *CmWRKY15-1* expression under stress treatments

Four-week-old seedlings were used to determine the expression patterns of *CmWRKY15-1* under different stress treatments. We sampled the leaves of cultivars C029 and Jinba at 0, 12, 24, 36, 48, and 72 h after treatment with *P. horiana* and at 0, 1, 6, 12, 24, and 48 h after treatment with 0.1 mM SA, 50 µM MeJA, 0.5 g L^−1^ ETH, and water. All the samples were stored at −80 °C, and each treatment was replicated three times. We quantified the relative expression levels of *CmWRKY15-1* via quantitative real-time PCR (RT-qPCR), with *CmActin* used as the internal control (with the primer pair CmActin-F/R). We performed real-time qPCR according to the instructions provided with SYBR^®^ Premix Ex Taq II. The PCR program was as follows: predenaturation at 95 °C for 30 s; 40 cycles of 95 °C for 5 s and 60 °C for 30 s; and a melt cycle of 95 °C for 15 s, 60 °C for 1 min, and 95 °C for 15 s. All the reactions were carried out three times for three independent biological replicates. We calculated the relative transcript levels of the target genes using the 2^−^^Δ^^Δ^^CT^ method^52^. We set the expression level of *CmWRKY15-1* in untreated leaves at 0–1 h for normalization across all the treatments.

### Construction of the transformation vector and genetic transformation

To construct the overexpression vector, we amplified the *CmWRKY15-1* full-length cDNA sequence via PCR after the addition of the enzymatic sites for XbaI and SacI using gene-specific primers (Supplemental Table 1). We purified the PCR product for ligation into a pTOPO vector to generate a p-TOPO-*CmWRKY15-1* construct, which was confirmed by sequencing, and digested it with XbaI and SacI to release the PCR product for subcloning into a pBI121 vector containing the cauliflower mosaic virus (CaMV) 35S promoter^53^. To generate an RNAi vector, pHANNIBAL was used as an intermediate vector. By using PCR, we amplified a 200-bp sense and a 200-bp antisense fragment from *CmWRKY15-1* containing the XhoI/KpnI and ClaI/HindIII restriction sites, respectively. The two fragments were digested with enzymes and then inserted into both sides of a *PDK* intron to yield a RNA hairpin construct. We then connected the hairpin RNA construct of pHANNIBAL to pBI121 to generate a *CmWRKY15-1* gene silencing vector. We subsequently introduced the pBI121-*CmWRKY15-1* overexpression construct and RNAi pRNAi-*CmWRKY15-1* construct into Agrobacterium (*Agrobacterium tumefaciens*) strain EHA105 and then separately transformed Jinba and C029 with pRNAi-*CmWRKY15-1*^54^.

### Confirmation of transgenic chrysanthemum

Putative transgenic plantlets of Jinba and C029 were rooted in Murashige and Skoog solid media supplemented with 0.2 mg L^−1^ NAA and 20 mg L^−1^ kanamycin. The positive plants were screened via PCR and RT-qPCR using the primers NptII-F/R and qRT-CmWRKY15-1-F/R (Supplemental Table 1).

### Phenotypic characterization of transgenic chrysanthemum

To determine the extent of disease resistance, we inoculated plants of the OE lines and RNAi lines and WT plants with 5–10 mL of a teliospore and basidiospore suspension. After 20 days, infection was observed and recorded for 30–40 leaves per line; the resistant types were determined and classified for each leaf according to the methods of Zhu^55^. We counted the number of blades per disease grade and then calculated the disease severity index (DSI) according to the following formula, based on the methods of Wang^56^: DSI = ∑(disease grade × number of blades) × 100% / highest disease level × total number of blades.

### Determination of endogenous salicylic acid levels

We sampled leaves from the WT, OE, and RNAi lines at 0 h and 24 h after pathogen infection. Plant extracts were prepared as described previously^57^. SA levels were then determined using a Plant Hormone Salicylic Acid ELISA Kit (ProNetsBio, Wuhan) according to the manufacturer’s instructions.

### Expression analysis of genes of *CmWRKY15-1* transgenic plants

We sampled the leaves of the OE lines, RNAi lines, and the wild type at 0 and 24 h after *P. horiana* inoculation. We quantified the relative expression of all the genes by RT-qPCR. The full list of primers used is provided in Supplementary Table S1.

### Statistical analysis

Three biological replicates were evaluated, with three technical replicates per biological replicate. All the data were analyzed using ANOVA and *t*-tests to determine significant differences with SPSS 24.0 software.

## Supplementary information

Supplementary Table 1
