# Supplementary material for: Chrysanthemum WRKY15-1 promotes resistance to Puccinia horiana Henn. via the salicylic acid signaling pathway
Source: Hortic Res. 2021 Jan 1;8:6. doi: 10.1038/s41438-020-00436-4 (PMC7775453; doi:10.1038/s41438-020-00436-4)
Supplement: Supplementary file 1 — Supplementary Table 1 [file 41438_2020_436_MOESM1_ESM.docx]

**Supplementary Table S1 Primers used in this study**

| Primer function | Primer name | Primer sequence (5′-3′) |
| --- | --- | --- |
| Cloned primers | CmWRKY15-1-1F  CmWRKY15-1-1R | ATGGTGGCTGCATCACAT  GGAAGAATCAGTGCTAATACATTAA |
| RT-qPCR primers | qRT-CmWRKY15-1-F  qRT-CmWRKY15-1-R | TAGGGCTTACTATAAGTGCTCTTTCG  CTTCCTTCCTGCTTTTGTGGTT |
| Reference gene primers | CmActin-F  CmActin-R | TCCGTTGCCCTGAGGTTCT  GATTTCCTTGCTCATCCTGTCA |
| pBI121-CmWRKY15-1 vector construction primers | CmWRKY15-1XbaI-F  CmWRKY15-1-SacI-R | CATTTGGAGAGAACACGGGGGACTCTAGAATGGTGGCTGCATCAC  GTTTGAACGATCGGGGAAATTCGAGCTCTTAACATACTTTGAATA |
| RNAi-CmWRKY15-1 vector construction primers | Sense-F | CCCTCGAGCGGGATCCGTGACGAGG |
|  | Sense-R | GGCCATGGGGAAGAATCAGTGCTAA |
|  | Ansense-F | CCATCGATGAAGAATCAGTGCTAAT |
|  | Ansense-R | CGAGCTCCCAAGCTTTGACGAGGTTTTGGTT |
| Selectable marker gene primers | NptII-F  NptII-R | GCTATGACTGGGCACAACAG  ATACCGTAAAGCACGAGGAA |
| SA synthesis genes | ICS1-F  ICS1-R | TCCCTACTGAAGAGGCACGG  CCAACAGCGGGTTCACTCTC |
|  | PAL-F  PAL-R | ATGGCACCGAAGCAAGTCACAC  GATACCCGAGTAACCCTGGAGGAG |
| Pathogenesis-related genes of the SA signaling pathway | NPR1-F  NPR1-R | TGTCGAGAAGGATGGAAAGCC  GGAGGCACCCATCATCAACA |
|  | PR1-F  PR1-R | CTCAACCAAAAGGAATAGTCGG  CCCTGCCAGTTTACGCTGTA |
|  | PR2-F  PR2-R | GGCAATGGTGGTGTTGGAAC  CTTCCTCCGTCAGCAGAAGG |
|  | PR5-F  PR5-R | CCAATGGAGTTTAGCCCCGT  GTCCACAACTACCACGCTCA |
